# *Fusarium*, an Entomopathogen—A Myth or Reality?

**DOI:** 10.3390/pathogens7040093

**Published:** 2018-11-28

**Authors:** Lav Sharma, Guilhermina Marques

**Affiliations:** CITAB—Centre for the Research and Technology of Agro-Environmental and Biological Sciences, University of Trás-os-Montes and Alto Douro (UTAD), Vila Real 5000–801, Portugal; gmarques@utad.pt

**Keywords:** beauvericin, entomopathogenic fungi, *Fusarium oxysporum*, *Fusarium solani*, insect biological control

## Abstract

The *Fusarium* species has diverse ecological functions ranging from saprophytes, endophytes, and animal and plant pathogens. Occasionally, they are isolated from dead and alive insects. However, research on fusaria-insect associations is very limited as fusaria are generalized as opportunistic insect-pathogens. Additionally, their phytopathogenicity raises concerns in their use as commercial biopesticides. Insect biocontrol potential of *Fusarium* is favored by their excellent soil survivability as saprophytes, and sometimes, insect-pathogenic strains do not exhibit phytopathogenicity. In addition, a small group of fusaria, those belonging to the *Fusarium solani* species complex, act as insect mutualists assisting in host growth and fecundity. In this review, we summarize mutualism and pathogenicity among fusaria and insects. Furthermore, we assert on *Fusarium* entomopathogenicity by analyzing previous studies clearly demonstrating their natural insect-pathogenicity in fields, and their presence in soils. We also review the presence and/or production of a well-known insecticidal metabolite beauvericin by different *Fusarium* species. Lastly, some proof-of-concept studies are also summarized, which demonstrate the histological as well as immunological changes that a larva undergoes during *Fusarium oxysporum* pathogenesis. These reports highlight the insecticidal properties of some *Fusarium* spp., and emphasize the need of robust techniques, which can distinguish phytopathogenic, mutualistic and entomopathogenic fusaria.

## 1. Introduction

*Fusarium* (Link ex Grey) species are hyaline filamentous fungi, which are ubiquitous with cosmopolitan distribution. They belong to the family Nectriaceae of the order Hypocreales within the fungal phylum Ascomycota. They can be found in air, water, plants, insects, soils and organic substrates. Based on host-associations, morphology and molecular characterizations, it is estimated that the genus *Fusarium* is comprised of at least 200 species recognized in 22 species complexes [1]. They are among the most destructive plant pathogenic and mycotoxigenic fungi. Their relevance in the agricultural industry is immense resulting in multi-billion dollar losses due to reduced crop yield. Moreover, they are reported frequently from infections in humans and animals. Some significant plant diseases caused include the *Fusarium* head blight in cereals, root rot in pea, ear rot in maize, sudden death syndrome in soybeans, and vascular wilts in numerous agricultural crops [2]. The prominence of *Fusarium* infections in plants can be estimated by the fact that two *Fusarium* spp., *Fusarium graminearum* Schwabe (teleomorph: *Gibberella zeae* (Schwein.) Petch) and *Fusarium oxysporum* Schlechtendahl are among the top five fungal pathogens of plants [3]. In animals, species such as *F. oxysporum* can cause infections in immunodepressed mice [4]. In humans, they cause infections collectively termed as fusariosis, which can be superficial such as keratitis or onychomycosis; locally invasive such as cellulitis, sinusitis or intertrigo; or deep or disseminated infections commonly occurring in severely immune-compromised patients [5]. Approximately 70% of *Fusarium* infections in humans are caused by *F. oxysporum* and *Fusarium solani* (Mart.) Sacc., the latter being more pathogenic and accounting for 50% of the infections [6]. Another species like *Fusarium moniliforme* Sheldon (*Fusarium verticillioides* (Sacc.) Nirenberg, or *Fusarium fujikuroi* Nirenberg; teleomorph: *Gibberella fujikuroi* (Sawada) Wollenw.) is relatively less prevalent, and accounts for 10% of human infections. *Fusarium* spp. causing superficial localized infections in humans as well as those which are phytopathogenic, were found pathogenic to insects, such as *Galleria mellonella* Linnaeus (Pyralidae: Lepidoptera). Both clinical and environmental strains of *Fusarium* spp. such as *F. oxysporum* and *F. solani* caused 100% of the mortalities of the insect larvae [7,8]. Moreover, the administration of anti-fungal agents like amphotericin B, which are effective against clinical *Fusarium* strains, also increased the survivability of the *Fusarium* infected larvae [8]. Such reports highlight the trans-kingdom pathogenicity of the *Fusarium* species.

Associations of fusaria with insects have been reported regularly during the last century. Wollenweber and Reinking reported 15 *Fusarium* isolates from insects [9]. Gordon collected approximately 9000 *Fusarium* isolates from the year 1932 onwards [10]. His isolates from the insects and other fungi together accounted for less than 1%, and those insect-associated or “insecticolous fusaria” belonged to 12 taxa and were collected from 14 insect species [10]. Li reported four *Fusarium* spp. from the insects-pest of rice in China [11]. Feng-Yan and Quing-Tao reported 180 *Fusarium* isolates from approximately 150 dead or diseased insects including spiders [12]. Insect orders such as Lepidoptera, Coleoptera, Homoptera, Hymenoptera and Araneida were found infected by fusaria [12]. Several studies reporting pathogenicity of *Fusarium* spp., either in laboratory or on-field natural mycoses, against different insect orders were reported during the three decades, between 1950 to 1980 (reviewed in [13]). Similar observations on the entomopathogenicity of fusaria were reported by Cladon and Grove [14].

Teetor-Barsch and Roberts suggested that using entomopathogenic fusaria could be advantageous in insect biological control, as some insect-pathogenic strains (a) demonstrate high host specificity; (b) can be easily grown in a laboratory; (c) do not damage plants; and (d) survive better as saprophytes in soils [13]. Nonetheless, studies in this area never achieved the required focus as *Fusarium* is a renowned plant pathogen, and using it as an insect biological control agent may lead to the release of phytopathogens into agroecosystems. O’Donnell et al. argued that *Fusarium* spp. might be used in insect biocontrol if entomopathogenic fusaria could be distinguished from phytopathogenic strains using genetic markers [15]. Insecticolous fusaria can be found in association with insects, either as a symbiont, opportunistic pathogen, saprophyte-eating decaying body, or as an entomopathogen. Here we briefly discuss studies, which focus on insect-*Fusarium* mutualism, and argue the entomopathogenicity of *Fusarium*, in detail.

## 2. Insect-*Fusarium* Mutualistic Association

In the past, some *Fusarium* spp. such as *Fusarium javanicum* Koord., *F. moniliforme*, *F. solani*, *Fusarium roseum* Link and *F. oxysporum* were reviewed for their non-pathogenic associations with many beetles and other insects [13]. Ambrosia beetles (Scolytinae, Curculionidae: Coleoptera) vector several fungal plant pathogens, which live inside the hosts as mutualists. *Euwallacea* Hopkins spp. are fungus farming beetles which cultivate mutualist fusaria. The majority of these beetles do not cause harm to living plants but instead attack decaying or recently killed trees. However, some of them colonize living tress and, along with *Fusarium* spp., can cause disease such as *Fusarium* canker or *Fusarium* dieback [16]. The fusaria belonging to ambrosia beetles generally belong to the *Fusarium solani* species complex (FSSC). Overall, FSSC includes more than 60 species and at least 12 of them belong to the *Fusarium* ambrosia clade, although 10 of these species have not been described yet. These insect-mutualist fusaria have adapted themselves for symbiosis, as their morphology is different than the free-living fusaria [17]. It was thought that the *Euwallacea*-*Fusarium* association was highly specific, but phylogenetic studies have demonstrated that this mutualism has experienced multiple symbionts shifts during the course of evolution [18]. 

Ambrosia beetles harbor these fungi within their special external structures known as “mycangia”. These beetles need these *Fusarium* spp. to digest lignocellulose and synthesize nutrients, such as ergosterols, which are required for pupation. There might be other roles of mutualist fusaria, however, it has been noted that, overall, the presence of fungi helps with insect growth and fecundity [19]. Occasionally, members of the FSSC have also been found associated with other beetles, such as the Asian longhorned beetles *Anoplophora glabripennis* Motschulsky (Coleoptera: Cerambycidae) [19]. Scully et al. performed a proteomic analysis of a *F. solani* isolate obtained from the gut of *A. galbripennis* grown on woody tissue, and found 400 expressed proteins, which are needed for plant cell wall degradation and digestion, and to recycle nitrogenous waste during periods of nutrient limitation [19]. These proteins encompassed several cutinases, pectate lyases, lipases, esterases, polysaccharide deacetylases, ureases, glycosyl hydrolases, laccases, peroxidases, and enzymes needed for hydrogen peroxide production, probably for lignin depolymerization [19]. 

## 3. *Fusarium* as an Entomopathogen

### 3.1. Isolation Studies Claiming Fusarium Entomopathogenicity

Hajek and Goettel suggested that the insect host range of an entomopathogen in a controlled laboratory environment is its “physiological host range”. Another term, i.e., “ecological host range” of an entomopathogen is the range of the insect species which can be infected in field conditions [20]. There have been some studies in the past that suggest the entomopathogenic potential of fusaria in a laboratory setting [21,22]. However, caged laboratory conditions provide an additional benefit to the pathogen, and it is not a surprise that the physiological host range is generally higher than the ecological host range. Therefore, to reduce any bias and to be sure about the entomopathogenicity of *Fusarium* spp., we undertook a stringent approach as described below, and reviewed only those studies where at least the species names of obtained fusaria were mentioned. In this section, the focus was on the investigations undertaken after the review by Teetor-Barsch and Roberts [13].

- Only those studies were considered where fusaria were found on the insects in field conditions, not on stored grains, and where Koch’s postulates for pathogenicity were confirmed.
- Moreover, those studies where fusaria were isolated from soils using an insect-bait were also considered, if Koch’s postulates were subsequently confirmed for the isolated strains.

#### 3.1.1. Isolations from Soils using the Insect-Bait Method and subsequent Koch’s Postulates Confirmation

Soil baiting using an infection sensitive insect such as the larvae of *G. mellonella* is a widely known technique for the isolation of entomopathogenic fungi from soils [23,24,25]. Generally this methodology is used to report entomopathogens such as *Beauveria* Vuillemin (Hypocreales: Cordycipitaceae) and *Metarhizium* Sorokin (Hypocreales: Clavicipitaceae). However, a few studies chose to mention the isolation of *Fusarium* spp. using this technique [25,26,27]. A total of seven *Fusarium* spp. exhibiting entomopathogenicity, were isolated from the soils, *Fusarium avenaceum* (Fr.) Sacc., *Fusarium heterosporum* Nees, *F. moniliforme*, *F. oxysporum*, *Fusarium semitectum* Berk. & Ravenel., *F. solani* and *Fusarium redolens* Wollenw. (Table 1).

#### 3.1.2. Natural On-Field Insect Mycoses and Subsequent Koch’s Postulates Confirmation

We carefully selected studies which claimed the entomopathogenicity of fusaria obtained from field infected insect cadavers. The criteria undertaken has already been mentioned earlier. A total of eight studies could meet this criteria and ten *Fusarium* spp. were found exhibiting natural entomopathogenicity, *Fusarium acuminatum* Ellis & Everh., *F. avenaceum, Fusarium culmorum* (Wm. G. Sm) Sacc., *Fusarium equiseti* (Corda) Sacc., *Fusarium merismoides* Corda, *F. oxysporum*, *Fusarium proliferatum* (Matsush.) Nirenberg ex Gerlach & Nirenberg, *Fusarium pseudograminearum* Aoki & O’Donnell, *F. solani* and *F. verticillioides* (Table 2).

### 3.2. Presence and/or Production of Insecticidal Metabolite beauvericin in Fusarium spp.

Beauvericin is a cyclic hexadepsipeptide belonging to the enniatin antibiotic family [28]. It is one of the active constituents of the entomopathogenic fungi *Beauveria bassiana* (Balsamo) Vuillemin (Hypocreales: Cordycipitaceae), and exhibits insecticidal, antimicrobial and anti-tumor activities. It was first isolated in 1969 from *B. bassiana* and *Isaria fumosorosea* Wize *(Paecilomyces fumosoroseus* (Wize) Brown & Smith) [29]. Later, it was also reported in other members of entomopathogenic fungal family Cordycipitaceae, such as *Isaria tenuipes* Peck and *Cordyceps cicadae* (Miq.) [30]. 

Gupta et al. reported the first isolation of beauvericin from *Fusarium* spp. [31], and later, a few studies such as Logrieco et al. [32] and, Stępień and Waśkiewicz [33] studied the production of beauvericin by *Fusarium* species. Moreover, some reports also reviewed the presence of beauvericin within *Fusarium* spp. [30,34]. The occurrence of beauvericin in *Fusarium* spp. and the members of Cordycipitaceae was so frequent that beauvericin was suggested as a chemotaxonomic marker for these fungi [30,34]. Due to the importance of beauvericin as an insecticidal metabolite, we also reviewed studies, which reported its isolation and/or the presence in the genome of *Fusarium* spp. At least 25 different *Fusarium* spp. produced beauvericin (Table 3).

Enniatins (ENNs) are also among the toxins belonging to the enniatin antibiotic family. They are produced by various *Fusarium* spp. and are reviewed by Jastoi [34]. The most prevalent ENNs analogs are ENN A, A1, B, and B1 [35]. Like beauvericin, these toxins also exhibit insect-pathogenicity. Numerous studies in the past have reported their entomopathogenicity [36,37,38]. Although it has been reported that fungi such as *F. avenaceum* and *Fusarium tricinctum* (Corda) Sacc. do not produce beauvericin, however, they produce significant amount of ENNs [39,40,41].

### 3.3. Proof-Of-Concept Studies on Fusarium (oxysporum) Entomopathogenicity

Although the occurrence of insecticolous fusaria is very common, there was not enough evidence to propose that *Fusarium* spp. were “real” entomopathogens, alike *Beauveria* or *Metarhizium*. In this direction, Navarro-Velasco et al. presented a detailed study based on histological evidence and dose-response curves highlighting the infectivity of *F. oxysporum* in *G. mellonella* larvae [7]. The study demonstrated: (a) The hyphal proliferation within the host hemocoel; (b) fungal interactions with host hemocytes; (c) progressive melanization; and (d) host colonization of the fungus. It was demonstrated that larval mortalities by *F. oxysporum* occurred through an active infection mechanism instead of a merely physical effect caused by the fungal conidia [7]. A similar study was conducted by Coleman et al. suggesting that the entomopathogenicity of fusaria fluctuated with varying temperatures and fungal conidial morphology [8]. Muñoz-Gómez et al. undertook a quantitative proteomics approach to identify the proteins and peptides involved in an elicited immune response in the hemolymph of *G. mellonella* larva infected with *F. oxysporum* microconidia [59]. Moreover, the importance of mitogen-activated protein kinases (MAPKs) of *Fusarium* has been demonstrated in its pathogenicity against insect larva [60]. In addition, a few studies have reported the presence of antifungal peptides after larval immunization by fusaria [61,62]. These studies are the proof-of-concept investigations, which demonstrate the entomopathogenicity of *Fusarium* spp. like *F. oxysporum*.

#### 3.3.1. Effect of Fungal Morphology and Ambient Temperature on Insect Mortality

Coleman et al. reported that both clinical and environmental fusaria are able to cause mortality among *G. mellonella* larvae. Larval moralities were noticed at 30 °C as well as 37 °C, however, larval killings were more rapid at 30 °C [8]. Conidial morphology is also vital and it was found that the fungal macroconidia were more virulent than the microconidia.

#### 3.3.2. Mitogen-Activated Protein Kinase (MAPK) Cascades: A Case of Key Fungal Pathways Responsible in Recognizing Cues during Insect Pathogenesis by *Fusarium* (*oxysporum*)

A fungal response to environmental and host cues is regulated by many functional biochemical pathways. Mitogen-activated protein kinase (MAPK) cascades are one of such pathways responsible in perceiving these molecular cues. The majority of the ascomycetous fungi, except *Saccharomyces cerevisiae* Meyen ex Hansen, possess only three MAPKs. These MAPKs are orthologous to *Fusarium* MAPK ((Fmk1) (or *S. cerevisiae* Fus3/Kss1)), yeast MAPK (Mpk1) and high-osmolarity glycerol MAPK (Hog1) [63]. The *Fusarium* MAPK, Fmk1, regulates infection-related morphogenesis and invasive growth, and Mpk1 is necessary for fungal cell wall remodeling and integrity. Another MAPK, Hog1, is crucial for adaptive response during hyperosmotic stress, besides mediating sensitivities to certain fungicides [60]. 

Segorbe et al. studied the contribution of these three MAPKs within *F. oxysporum*, with respect to the development, virulence and stress response in the insect-host *G. mellonella* [60]. It was found that strains with Mpk1 or Hog1 deletions showed a significant decrease in virulence in comparison with the wild type strain. In addition, virulence was further decreased significantly in the double mutant strains with Mpk1 and Hog1 deletions, when compared to single deletion mutants of these MAPKs. This suggests that Hog1 and Mpk1 have distinct and additive virulence functions in *F. oxysporum* pathogenesis in *G. mellonella* [60].

#### 3.3.3. Production of Antimicrobial Peptides in Insect Larva Post *Fusarium* Immunization

A few studies have investigated the presence of antifungal peptides in the hemolymph of immune stimulated *G. mellonella* larva. Brown et al. reported moricin-like peptides in the hemolymph of immune stimulated *G. mellonella* larva [61]. These peptides exhibited in vitro inhibition of *F. oxysporum* and *F. graminearum*. In another study, an increase of *Galleria* defensin, lysozyme, and proline-rich peptide 2 was observed in *G. mellonella* hemolymph after immunization by *F. oxysporum* [62]. The maximum concentrations of these peptides were noticed 72 h post-immunization.

#### 3.3.4. Induced Protein Expression in Insect Larva during *Fusarium* (*oxysporum*) Infection

Muñoz-Gómez et al. studied the proteins which were expressed differentially within the hemolymph of *G. mellonella*, in the control and immunized larval sets, when two different concentrations of *F. oxysporum*, i.e., 10^4^ or 10^6^ microconidia/mL were injected. The study also investigated the changes in immune response when the temperature was raised from 25 °C to 37 °C. They observed an expression change of over 50 proteins, and 17 of them were supposed to be involved in the insect’s immune response [59]. Here we reviewed some of those immune response related proteins which got differentially regulated at 25 °C. We also briefly discuss the general role of those proteins in insects.

##### Crucial Down-Regulated Proteins

Arylphorin and Apolipophorin, which were supposed to be quite abundant in the hemolymph, were down-regulated after *F. oxysporum* invasion [59]. Ayrlphorins is a class of ~500 kDa glycoproteins which are produced by the insect larva as reserves during the metamorphosis and egg development stages [64]. Apolipophorins help in lipid transport and are thought to be associated with antimicrobial activities, as lipids are secreted to prevent a microbial invasion. In particular, Apolipophorin III was found to be the most down-regulated protein in the study by Muñoz-Gómez et al. (2014) [59]. This protein increases the antibacterial activity within the hemolymph, and the production of superoxide by the hemocytes [65]. Muñoz-Gómez et al. hypothesized that it may stimulate antimycotic activities within the hemolymph. Another glycoprotein, i.e., larval hemolymph protein was also found to be down-regulated [59]. 

Hexamerins were also found to be down-regulated. They are regularly secreted by the larval fat body and stored within the hemolymph, and during metamorphosis they return to the larval fat body to be processed [66]. Transferrin removes the essential iron ions during pathogen invasion and makes the insect hemocoel unsuitable for microbial colonization [67]. These proteins undertake antibacterial iron-withholding strategies within the insects [68]. Muñoz-Gómez et al. found that the protein Transferrin was also down-regulated which, according to them, act as an antimycotic mediator enhancing insect survival by preventing oxidative stress [59].

##### Crucial Up-Regulated Proteins

Cationic protein 8 precursor was also found to be an abundant protein which was upregulated extraordinarily [32]. Kim et al. reported that it functions as an opsonin, which promotes the uptake of invading microbes into hemocytes, and therefore, contributing in phagocytosis by hemocytes [69]. Another up-regulated protein, i.e., Hemolin, is a member of the immunoglobulin protein superfamily, which interacts in lepidopteran insects as an opsonin. Hemolin is associated with hemocytes, and as an opsonin, it facilitates recognition of pathogens and mediates hemocytic immune response [70,71,72]. Increased Hemolin can be seen as an insect response towards mycotic infection as its expression was always up-regulated when microconidia concentration was increased [59]. 

Lysozyme was also up-regulated, which is a well-known anti-bacterial enzyme, and is cold-adapted to work in insects dwelling at lower temperatures [73]. Melanization is an important physiological change occurring inside hemocoel when an insect is invaded by a pathogen, for example, *Beauveria*. Interestingly, Lysozymes have also been reported for its anti-melanization activity inside mosquitoes [74]. 

During an infection process, the immune cells migrate, adhere and phagocytose invading pathogens, and contribute to wound repair. Hence, cell adhesion and phagocytosis are quite critical in eliminating infection-causing microbes. These two processes depend on the dynamics of the Actin network, i.e., Actin filaments polymerization and depolymerization [75]. Up-regulation of Actin during *F. oxysporum* invasion of *G. mellonella* larva further highlights the entomopathogenicity of the fungus [59].

## 4. Safety and Side Effect of Using Entomopathogenic Fusaria on Plants, and Possible Genetic Attunements Facilitating Use as a Biopesticide

Studies focusing on the safety of using insect-pathogenic fusaria on plants showed mixed results. Some previous investigations have documented their safety in terms of fungal application on plants. An investigation was conducted to test the infectivity of *F. oxysporum* isolated from the brown planthopper, *Nilaparvata lugens* Stål (Hemiptera: Delphacidae) on the rice, cotton and tomato plants. Four different fungal treatments, i.e., soil, root, seed and leaf, were used to test the possibility of an infection. However, the results were negative in all of the cases and the entomopathogenic strain was found safe for the plants [76]. In another study, conidial sprays of *Fusarium* on grape phylloxera *Daktulosphaira vitifoliae* Fitch (Hemiptera: Phylloxeridae) reduced insect populations encompassing different life stages ranging from eggs, larva and adults [77]. Sprayed *Fusarium* was found to be a saprophyte associated with grape leaves and caused no infections in the plant [77]. 

On the contrary, a few studies reported the infectivity of entomopathogenic fusaria on plants. Strains of *F. oxysporum*, *F. solani* and *F. roseum* from *Sitona hispidula* Fabricius and *Sitona flavescens* Marsh (Coleoptera: Curculionidae) were also found pathogenic to these larvae. However, isolated *F. oxysporum* exhibited pathogenicity to clover *Trifolium pretense* L. [78]. 

To access the infectivity of entomopathogenic *F. oxysporum* on tomato plants, Navarro-Velasco et al. studied the effects of different fungal mutant strains on the plant [7]. Deletion of different loci led to distinct results in terms of their pathogenicity towards the tomato plant and *G. mellonella* larval killings. Mutants without the locus *fmk1* [79], the heterotrimeric G protein β-subunit (*fgb1*) [80], or β-1,3-glucanosyltransferase *(gas1*) [81] exhibited marginal or no infectivity to the tomato plant, however, all these strains exhibited considerable entomopathogenicity. Hence, Navarro-Velasco et al. demonstrated that through specific mutations, phytopathogenicity of *Fusarium* strains can be checked and they can be attuned for biological control of insects [7]. 

## 5. Conclusions

*Fusarium* spp. has long been thought of as an opportunistic insect pathogenic fungi, or saprophytes, which colonizes decaying bodies of insects. Furthermore, as fusaria are well-known plant pathogens, such generalizations only grow stronger. With increasing evidence of in-field insect mycoses by fusaria, it’s time to reconsider its biological function besides being phytopathogens and saprophytes. Studies summarized within this review range from ecological to experimental and immunological, and will help researchers to gain insights into the role of fusaria as entomopathogens. It was noted that fusaria could exhibit mutualism with insects, however, this property was generally restricted to strains belonging to the *F. solani* species complex. More proof-of-concept studies on insects from different orders will further strengthen the claims of the entomopathogenicity of different *Fusarium* species. It has been observed that entomopathogenic fusaria can be safe for plants. Furthermore, phytopathogenicity of entomopathogenic fusaria can be attenuated through deletions of a few loci. We urge for the development of robust markers which could distinguish fusaria that solely exhibit entomopathogenicity. Additionally, we stress on the need for molecular studies differentiating between the fungal genetic loci solely used in entomopathogenicity, from those imparting phytopathogenicity in fusaria. Such studies would facilitate the development of a few *Fusarium* strains as biological control agents of some insect-pests of interest.

## Figures and Tables

**Table 1:** Soil isolations of *Fusarium* spp. using *Galleria*-bait method and subsequent pathogenicity confirmation.

*Fusarium* Species	Isolation Numbers and Respective Frequencies	Koch’s Postulate Confirmed (A *)	Percentage (%) Mortality During A *	Plantation	Country	Reference
*F. avenaceum*	11 (2.91%)	yes	0–26.7	Forests	China	[27]
*F. heterosporum*	1 (1.4%)	yes	18	Citrus orchard	Palestine	[26]
*F. oxysporum*	35 (9.3%)	yes	0–93.3	Forests	China	[27]
	2 (2.9%)	yes	30–33	Vegetable fields and citrus orchard	Palestine	[26]
*F. solani*	5 (7.14%)	yes	28–44	Vegetable fields and citrus orchard	Palestine	[26]
	18 (4.8%)	yes	0–86.7	Forests	China	[27]
*F. redolens*	1 (0.26%)	yes	26.7	Forests	China	[27]
*F. semitectum*	7 (10%)	yes	16–33	Vegetable fields	Palestine	[26]
*F. verticillioides* *(=F. moniliforme* *or F. fujikuroi;* teleomorph: *Gibberella fujikuroi)*	1 (1.4%)	yes	30%	Vegetable fields	Palestine	[26]

* In the studies mentioned above, a quick dip or touch on fungal conidia sporulating on insects were used for pathogenicity confirmation.

**Table 2:** On-field natural insect mycoses by *Fusarium* spp. and the pathogenicity confirmation of the obtained isolates.

*Fusarium* Species	Insect-Host	Number of Fungal Isolates and/or Occurrence Frequencies	Koch’s Postulate Confirmed (A)	Quantity of Fungal Concentration Used in A	Percentage Mortality during A	Plantation	Country	Reference
*F. acuminatum*	*Cephus cinctus* Norton (Hymenoptera: Cephidae)	8 (3.7%)	Yes, with one isolate	1.5 × 10^4^–1.5 × 10^8^ conidia/mL	~60% at 1.5 × 10^8^ conidia/mL	Wheat	USA	[42]
*F. avenaceum*	*C. cinctus*	27 (12.4%)	Yes, with one isolate	1.5 × 10^4^–1.5 × 10^8^ conidia/mL	~90% at 1.5 × 10^8^ conidia/mL	Wheat	USA	[42]
*F. culmorum*	*C. cinctus*	126 (58.1%)	Yes, with one isolate	1.5 × 10^4^–1.5 × 10^8^ conidia/mL	~80% at 1.5 × 10^8^ conidia/mL	Wheat	USA	[42]
*F. equiseti*	*C. cinctus*	31 (14.3%)	Yes, with one isolate	1.5 × 10^4^–1.5 × 10^8^ conidia/mL	~80% at 1.5 × 10^8^ conidia/mL	Wheat	USA	[42]
*F. merismoides*	*P. quercivorus*	5 (17.86%)	yes	n/a	7.17%	Oak logs	Japan	[43]
*F. oxysporum*	*Brahmina coriacea* Hope (Coleoptera: Scarabaeidae)	262 (18.66%)	yes	n/a	49.63%	Potato	India	[44]
	*Platypus quercivorus* Murayama (Coleoptera: Platypodidae)	4 (14.28%)	yes	n/a	53.93%	Oak logs	Japan	[43]
	Insects from the orders Homoptera, Coleoptera and Lepidoptera.	246 (70.29%)	Yes, using one isolate	10^8^ conidia/mL	97.5%	Chilli, Palo de rosa plant and Maize	Mexico	[45]
	*Planococcus ficus* (Signoret) (Hemiptera: Pseudococcidae)	1 (4.55%)	Yes	10^8^ conidia/mL	50%	Vines	Portugal	[46]
*F. proliferatum*	*Dryocosmus kuriphilus* Yasumatsu (Hymenoptera: Cynipidae)	2 (3.55%)	yes	2 × 10^6^ conidia/mL	33% and 99%	Chestnut	Italy	[47]
*F. pseudograminearum*	*C. cinctus*	25 (11.5%)	Yes, with one isolate	1.5 × 10^4^–1.5 × 10^8^ conidia/mL	>95% at 1.5 × 10^8^ conidia/mL	Wheat	USA	[42]
*F. solani*	*B. coriacea*	120 (8.6%)	yes	n/a	42.59%	Potato	India	[44]
	*P. quercivorus*	1 (3.5%)	yes	n/a	47.33%	Oak logs	Japan	[43]
	*Tetanops myopaeformis* Röder (Diptera: Ulidiidae)	44%	yes	>10^6^ conidia/mL	Considered pathogenic only after Koch’s postulate verification	Wheat during collection and Sugarbeet in the previous year	USA	[48]
	*Planococcus ficus* (Signoret) (Hemiptera: Pseudococcidae)	2 (9.10%)	Yes	10^8^ conidia/mL	45%	Vines	Portugal	[46]
*F. verticillioides* (=*F. moniliforme* or *F. fujikuroi*; teleomorph: *Gibberella fujikuroi*)	Insects from the orders Homoptera, Coleoptera and Lepidoptera.	100 (28.57%)	Yes, using one isolate	10^8^ conidia/mL	96.6%	Papaya and Maize	Mexico	[45]
	*Ceratovacuna lanigera* Zehntner (Homoptera: Aphididae)	Total four strains isolated	Yes; one isolate was re-tested in field	10^7^ CFU/mL for Koch’s postulate; 10^8^ CFU/gm powder for the field	60% effective mortality in field	Sugarcane	India	[49]
	*Tropidacris collaris* Stoll (Orthoptera: Acridoidea)	One strain isolated for further testing	Tested on another insect	2.8 × 10^6^ conidia/mL	58% against *Ronderosia bergi* Stål (Orthoptera: Acrididae)	Dense woodland	Argentina	[50]
	*Planococcus ficus* (Signoret) (Hemiptera: Pseudococcidae)	1 (4.55%)	Yes	10^8^ conidia/mL	40%	Vines	Portugal	[46]

**Table 3:** Studies reporting the production and/or presence of genetic loci in *Fusarium* spp. encoding for the insecticidal metabolite beauvericin.

*Fusarium* Species	Report of Genes, or Presence in Genome	In-Vitro or In-Vivo Production	References
*F. acuminatum*	Y	Y	[32,33,51]
*F. ananatum*	Y	Y	[33,52]
*F. anthophilum*	Y	Y	[32,33]
*F. beomiforme*	−	Y	[32,52]
*F. circinatum*	Y	Y	[52,53]
*F. concentricum*	Y	Y	[33]
*F. dlamini*	−	Y	[32]
*F. equiseti*	−	Y	[32]
*F. longipes*	−	Y	[32]
*F. mangiferae*	Y	Y	[53]
*F. nygamai*	Y	Y	[32,33]
*F. oxysporum*	Y	Y	[32,33]
*F. poae*	Y	Y	[32,33,39,54]
*F. proliferatum*	Y	Y	[33,55,56,57]
*F. redolens*	−	Y	[51]
*F. sambucinum*	−	Y	[32]
*F. semitectum*	−	Y	[31]
*F. sporotrichioides*	−	Y	[33,39,41]
*F. subglutinans*	Y	Y	[32,33]
*F.* *temperatum*	Y	Y	[33]
*F. glubosum*	−	Y	[56]
*F. guttiforme*	−	Y	[52]
*F. konzum*	−	Y	[58]
*F. pseudoanthophilum*	−	Y	[52]
*F. verticillioides* (=*F. moniliforme* or *F. fujikuroi*; teleomorph: *Gibberella fujikuroi*)	Y	Y	[33,48,57]

Note: Y signifies yes and the minus (−) sign signifies ‘no report yet’.
